# Single intratesticular injection of blood‐serum‐derived exosomes can potentially alleviate testopathy following testicular torsion

**DOI:** 10.1002/ame2.12232

**Published:** 2022-05-20

**Authors:** Mona Keivan, Fatemeh Mansouri Torghabeh, Samira Davoodi, Shima Moradi Maryamneghari, Reza Dadfar

**Affiliations:** ^1^ Member of Research Committee, Medical School Kermanshah University of Medical Sciences Kermanshah Iran; ^2^ Department of Physiology Sciences, Medical School Mashhad University of Medical Sciences Mashhad Iran; ^3^ Department of Anatomical Sciences, Medical School Kermanshah University of Medical Sciences Kermanshah Iran; ^4^ Department of Anatomical Sciences, Faculty of Medicine AJA University of Medical Sciences Tehran Iran

**Keywords:** anti‐inflammation, exosome, parameter, sperm, testicular torsion

## Abstract

**Background:**

Testicular torsion (TT) is an acute inflammatory process leading to male infertility. Today, anti‐inflammatory effects of exosomes derived from blood serum are used in various laboratory procedures. In the present study, the anti‐inflammatory effects of blood‐serum‐derived exosomes in treatment of acute inflammation following TT in mice were evaluated.

**Materials and Methods:**

Eighteen male mice were grouped as healthy control, TT, and TT + exosome. TT was induced surgically, and exosomes were extracted from blood serum and administrated by a single intratesticular injection (10 IU). Malondialdehyde (MDA) and Griess assays were used to evaluate the level of oxidative stress. Sperm indices, testosterone (Tes), and apoptotic gene expression (p‐53, Bcl2, and Caspase‐3) were also assessed. H&E and immunohistochemistry (IHC) stainings were used for histopathological investigations. Data analysis was applied by SPSS (v.19) software.

**Results:**

Oxidative stress and apoptotic genes expression were increased significantly (*p* < 0.05) in TT group compared with control. Sperm parameters and Tes were significantly increased, and expression of apoptotic genes was significantly reduced in TT + exosome group (*p* < 0.05).

**Conclusion:**

Since the blood‐serum‐derived exosomes have anti‐inflammatory features, the intratesticular application of blood‐serum‐derived exosomes can be used clinically in acute phase of orchitis following TT to inhibit testicular inflammation.

## Graphical abstract

Blood‐derived exosomes can act as anti‐inflammatory sources in TT. This study showed that administration of a single dose of exosome immediately after an acute testicular injury can significantly reduce TT complications. Our study showed that exosomes can diminish apoptosis and testicular inflammation and lead to the production of high‐quality sperm. It is suggested that in other experimental studies, the molecular effect of exosomes in the treatment of acute inflammation should be investigated to determine the apparent role of this substance. Thus, the exosome may be suggested as a treatment protocol for the relief of acute inflammation such as TT. For future investigations, it is recommended to use exosomes derived from other body fluids in TT pathologic condition.

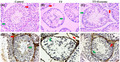

## 1 INTRODUCTION

The sexual system in men includes the testicles, seminal vesicles, and penis. The most important part of this system is the testicles, which produce sperm and the hormone testosterone. The testicles are located inside the sac and are protected by different layers. The structure of the testicles consists of the intricate tubes that make up sperm. The intermediate layers of the seminiferous tubules are composed of different densities of Leydig cells. Damage to the fallopian tubes leads to the cessation of sperm production, and damage to Leydig cells leads to a decrease in testosterone production and thus the elimination of secondary sexual characteristics in men. Testes are involved in 2 primary physiological processes: spermatogenesis and sexual hormone secretion. Healthy sperms are able to fuse with the egg successfully during the process of fertilization. Thus, the preservation of the healthy status of sperms seems a critical issue. Testes with physiological Tes production and healthy sperm generation are considered normal sexual tissue, whereas inflammation of testis can lead to pathological disruption of sperm production or hormone secretion.^1^

TT is a common clinical emergency in men. In TT, the testis rotates around the longitudinal axis of the testicle, leading to artery compression and blood stasis.^2^ These conditions can lead to testicular inflammation. Inherently, the testes are sensitive to toxins and blood homeostasis.^2^ Following orchitis, the agents with oxidative features accumulate in testicular tissue with inhibitory effects on functions of Leydig and spermatogenic cells. Thus, in chronic status, Tes secretion and normal spermatogenesis can be affected negatively with expression of inflammatory genes and intercellular secretion of cytokines and interleukins.^3^, ^4^ In this status, the expression of cell death genes can be elevated, leading to extensive apoptosis in seminiferous tubules and Leydig cells. Administration of exogenous anti‐inflammatory agents can inhibit orchitis, leading to the restoration of physiological activity of tissue.

Exosomes are membrane‐bound vesicles that are present in body fluids. Numerous studies have approved the effect of exosomes on restoration of physiological processes. Today, the majority of the new therapeutic treatments are based on the extraction of exosomes from different body fluids and their applications by different protocols.^5^ It seems that, through exosome extraction from the body fluids, the challenge of autoimmune reaction will also be eliminated. Numerous articles have referred to the anti‐inflammatory effect of exosomes. Thus, we assumed that application of anti‐inflammatory agents such as exosomes could reduce inflammation and increase cell regeneration.^6^

Since the anti‐inflammatory features of many types of exosomes have been reported in various papers, this animal study was designed to experimentally evaluate the role of serum‐derived exosomes on inhibition of inflammation caused by TT. Various laboratory assays were employed to measure the oxidative levels following TT, such as Griess and MDA. Also, to observe the probable therapeutic effects of exosomes on tissue regeneration, immunohistochemistry (IHC) and routine H&E stainings were used, and gene expression was measured.

## 2 MATERIALS AND METHODS

### 2.1 Experimental animal characteristics

Following preparation of 18 male adult mice (NMRI, 8–12 weeks, 20–25 g), standard living conditions were provided, including normal environmental temperature, 50%–60% humidity, physiological photocycle, and free access to food and water. All experimental manipulations were conducted in accordance with ethical principles described in an appropriate version of the 1975 Declaration of Helsinki (revised in 2000).

### 2.2 Exosome isolation from blood serum

Blood serum was used as a source of exosome isolation using the Exosome Purification Kit (Exo‐spin TM, 8 columns, Cell Guidance System, Cat EX01). This kit contained; 2× Exo‐spin Buffer, 8× Exo‐spin columns with waste collection tubes, and 1× PBS (7 ml) without calcium chloride and magnesium chloride. This kit combines precipitation and size exclusion chromatography (SEC) techniques to inhibit the isolation of non‐exosomal materials. Just prior to exosome purification, the blood samples were centrifuged (4000 rpm, 15 min), and blood serum was used for exosome isolation to preserve the high quality of purified exosomes. For debris elimination, 1 ml of plasm was transferred to a microcentrifuge tube and treated by centrifugation (room temperature, 500 rpm, 10 min). This protocol was also repeated for the supernatant (12 000 rpm, 30 min) to remove probable remaining cell particles. For precipitation of exosome‐containing fractions, the isolated supernatant was transferred to a new microcentrifuge tube and received Exo‐spin Buffer (with a buffer:supernatant ratio of 1:2). This solution was preserved in a refrigerator (4°C) for 60 min (12 000 rpm). The supernatant was removed, and the palette was resuspended in 100 μl of PBS. Exo‐spin column matrix was prepared and re‐equilibrated twice with 250 μl of PBS prior to application. The liquid was allowed to penetrate into the column matrix under gravity, and then the flow‐through buffer was discarded. For purification of exosomes, 100 μl of resuspended exosome‐containing pellet was added to the column (column was located in a collection tube). Flow‐through was discarded, and the column was placed into a new 1.5 ml microcentrifuge collection tube. Then, 180 μl of PBS was added to the column and was allowed to elute. Exo‐spin column was removed from the sample collection tube. Finally, the sample collection tube containing the isolated exosomes was centrifuged (1000 rpm, 30 s) to collect all liquid at the bottom of the tube. Also, no dilution was performed on the resulting product, and a solution containing exosome was injected into the testis (on the involved torsion‐detorsion side) with the volume of 10 IU.^7^

### 2.3 TT modeling

For anesthesia induction, 50 IU of ketamin 10 IU/xylazine 90 IU was injected intraperitoneally for in the animals. The left inguinal canal was dissected, and the spermatic cord was exposed. According to the routine procedure of Shahedi et al., the testis was rotated (270°) counterclockwise for 2 h (the anesthetic drug injection was extended every 30 min to continue the anesthesia for 2 h). In this period, the spermatic cord was fixed to the walls of inguinal canal using silk thread (4/0). Thus, blood flow was arrested, and orchitis was induced. Finally, all surgical conditions were restored to their anatomical position, and the walls of inguinal canal were sutured. ^8^

### 2.4 Study groups and drug administration

After 2 weeks of adaption of animals to the new environment, they were categorized into 3 groups (*n* = 6 in each group) including; (1) healthy control (healthy mice with no experimental manipulations), (2) TT (TT‐induced animals with no exosome administration), and (3) TT + exosome (TT‐induced animals received an intratesticular injection of exosomes). Four hours after TT, a single dose of exosome was injected in scrotum (10 IU). In this procedure, the skin of scrotum was elevated and the niddle was inserted subcutaneously. Thus, in this process, the drug was accumulated in subcutaneous tissue surrounding the testis.

### 2.5 Tissue sampling

A week after exosome injection, the samples were collected. Initially, anesthesia was induced, and the animals were killed by amputation of cervical vertebrae. Venous blood was drawn from the right ventricle and centrifuged to collect blood serum (4000 rpm, 15 min). The left testicle was also dissected in 2 identical parts for histopathological (placed in a 10% formaldehyde container for 3 weeks for tissue fixation) and genetical (frozen in liquid nitrogen) assessments. Also, the tail of the left epididymis was cut in 10 ml of DMEM F12/FBS 5% culture medium for sperm parameter assessments.^9^

### 2.6 Sperm indices examinations

To evaluate sperm quality, indices of viability, motility, count, and morphology were examined microscopically. Following preparation of cell suspension in previous step, 40 μl of eosin was combined with the same volume of cell suspension. Thus, dead cells were identified, and living sperms were counted using a microscope (40×). According to the Zamir‐Nasta protocol, the normal motility of sperms was assessed, and just the sperms with straight movement were considered to have normal sperm motility. For counting, 400 μl of fixed sperm suspension (using formaldehyde 10%) was located on a hemocytometer. Also, eosin/nigrosin was used to assess sperm morphology (400×).^10^

### 2.7 Griess technique

Concentration of tissue nitric oxide (NO) can be considered as one of the factors expressing tissue damage. NO was measured using an ELISA kit (Griess reagent, Abcam, ab234044). Following measurement of various dilutions containing NO at 540 nm wavelength, the standard curve was drawn, and the results are shown as optical density (OD).^11^

### 2.8 MDA assay

After tissue damage and cell death, the cell membrane lipids are peroxidized, and the resulting fragments are released in the form of thio‐barbituric acid reactive substances (TBARS). Therefore, the TBARS measurement using the MDA kit (MDA Assay Kit, Colorimetric/Fluorometric, ab118970) can be a criterion for reporting the amount of cellular peroxidation. Following preparation of the associated substances, including lysis buffer, phosphotungstic acid, and BHT (100×), the OD was measured at 532 nm. MDA concentration was measured using the following formula; the amount of MDA (nM)/mg × 4* × D = nM/mg. 4* was the correction for using 200 μl of the 800 μl reaction mix.^12^

### 2.9 Serum levels of Tes

Tes level is an important index representing the status of Leydig cell damage after TT induction. Also, this index shows the level of repairment following exosome administration. An ELISA kit (Abcam, cat. no. 108666) was employed to measure the Tes levels in blood serum using the routine procedure of Asghari et al.^13^

### 2.10 Histopathological H&E staining

After fixation of the samples in 10% formalin (for 3 weeks), tissue processing was performed (dehydration using increased concentrations of ethanol, clearing using xylene, wax infiltration using paraffin, and tissue embedding) was applied. Then, tissue sections (5 μm) were prepared using a microtome devise. H&E staining was used for histopathological assessments of slides according to various protocols of deparaffinization, hematoxylin staining, alcoholic‐eosin staining, acid‐alcoholic administration, and xylene‐based clearing. Seminiferous tubule, interstitial tissue, lumen, and spermatogenic cell lines were observed microscopically (40×).^14^

### 2.11 IHC staining of NF‐kB protein

NF‐kB proteins belong to a family of structurally related eukaryotic transcription factors involved in controlling a large number of physiological cellular and organismal processes, such as immune and inflammatory responses. Histological sections were incubated in methanol solution with H_2_O_2_ (3%). Sodium citrate buffer was used for antigen retrieval (pH 6, 10 mM, 98°C, 15 min). These retrieved slides in a 4°C environment were incubated by mice monoclonal antibodies against Bcl‐2 protein as the primary antibody. After one night, the sections were incubated with biotinylated goat anti‐mouse IgG. Then, they were exposed to 3,3‐diaminobenzidine substrate as chromogen for 8 min. Bounded antibodies appeared in brown.^15^

### 2.12 RNA extraction and real‐time quantitative PCR

Total RNA was extracted from the tissue of the left testis. Then, the quality was examined using a Nanodrop machine at 260/280 nm. Finally, the cDNA was synthesized, and real‐time quantitative PCR was conducted. The primers of related genes were p53 (F: *CCTCAGCATCTTATCCGAGTGG*, R: *TGGATGGTGGTACAGTCAGAGC*), Caspase‐3 (F: *GTGGAACTGACGATGATATGGC*, R: *CGCAAAGTGACT GGATGAACC*), and BCL‐2 (F: *CTAGCAAAGTAGAAGAGGGCAACC*, R: *TGTGGATGACTGACTACCTGAACC*) were evaluated. Also, β‐actin (F:*GGCACCACACCTTCTACAATG*, R:*GGGGTGTTGAAGGTCTCAAAC*) was used as a housekeeping gene. Gene expression was measured by using the 2^−ΔΔCt^ method.^16^

### 2.13 Statistical analysis

SPSS software (v. 16) was used for data analysis, and the final graphs were drawn using GraphPad Prism software. Results are expressed mean ± standard deviation (SD), and *p* < .05 was considered significant. Kolmogorov–Smirnov was used to assess the normal distribution of data. One‐way analysis of variance (ANOVA) was used for statistical analysis, and the Tukey post hoc test was used to determine the difference between the groups.

## 3 RESULTS

### 3.1 Sperm parameter assessment

All normal sperm indices were decreased significantly (*p* < .05) following TT induction in the TT group compared with the control. These features were alleviated significantly (*p* < .05) after exosome administration in the TT + exosome group compared with the TT animals (Table 1).

**TABLE 1:** Effect of TT and Exosome administration on sperm parameters

Group	Normal morphology (%)	Count (10^6^)	Motility (%)	Viability (%)
Control	38.2 ± 0.5	74 ± 2.9	21.13 ± 2.7	78.76 ± 6.14
TT	12.02 ± 0.3^*^	28.4 ± 3.6^*^	6 ± 0.3^*^	43.4 ± 2.7^*^
TT + exosome	32.54 ± 0.7^**^	68.12 ± 3.94^**^	20.6 ± 1.3^**^	66.14 ± 3.04^**^

*Note*: Data are presented as mean ± SD. *N* = 18 animals in 3 different groups.

Abbreviation: TT, testicular torsion.

^*^ *p* < .05 compared with the control group

^**^ *p* < .05 compared with the TT group.

### 3.2 Serum levels of NO

Following induction of TT, the NO levels showed a significant (*p* < .05) incremental trend in TT groups compared with the control animals. This index was decreased significantly (*p* < .05) in the TT + exosome group compared with TT animals after exosome injection (Table 2).

**TABLE 2:** Effect of TT and exosome administration on various serological parameters in male mice

Group	NO (OD)	Tes (ng/ml)	MDA (nm/gKW)
Control	28.5 ± 1.2	9.5 ± 1.3	61.84 ± 0.52
TT	48^*^	2.33 ± 0.6^*^	114.1 ± 2.54^*^
TT + exosome	32.3 ± 1.8^**^	6.8 ± 0.4^**^	93.3 ± 5.3^**^

*Note*: Data are presented as mean ± SD. *N* = 18 animals in 3 different groups.

Abbreviations: MDA, malondialdehyde; NO, nitric oxide; OD, optical density; Tes, testosterone; TT, testicular torsion.

^*^ *p* < .05 compared with the control group

^**^ *p* < .05 compared with the TT group.

### 3.3 Serum level of androgen

The level of Tes in blood serum of TT animals was decreased significantly (*p* < .05) compared with control. This androgen was also increased approximately similar to normal level following exosome administration in TT + exosome animals compared with the TT group (Table 2).

### 3.4 Serum level of MDA

After TT induction, the MDA level increased significantly (*p* < .05) in TT animals compared with control. This index was also significantly decreased in the TT + exosome group compared with TT animals (*p* < .05) (Table 2).

### 3.5 H&E and IHC stainings of seminiferous tubules

**FIGURE 1:**
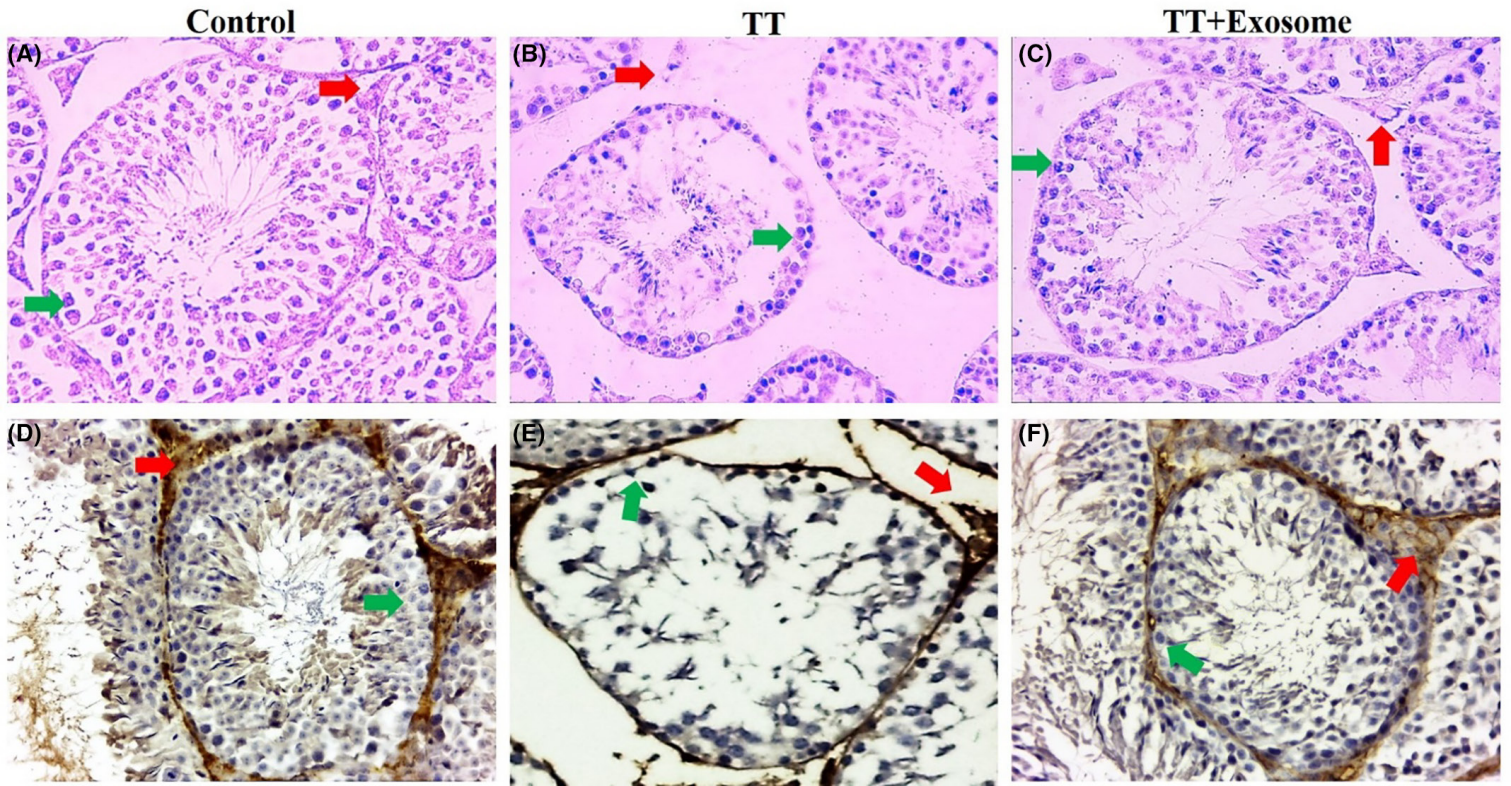
Histopathological H&E and IHC images of testis in control (A, D), TT (B, E), and TT + exosome (C, F) groups. Red arrow shows the interstitial connective tissue containing Leydig cell, and green arrow shows basal spermatogenic cell lines. 100× magnification. *N* = 18 animals in 3 different groups. H&E, hematoxyline and eosin; IHC, immunohistochemistry

Microscopic examination of H&E‐stained images showed that TT could lead to acute tissue damage to testicular tissue (Figure 1B). TT temporarily blocked the blood flow, causing tissue oxidation. This study showed that the layers of the spermatogenic lineage were highly reduced (Figure 1B). Besides, the basal germ cells maintained their presence (Figure 1B, green arrow). These changes lead to a temporary reduction in spermatogenesis, but eventually, the damage appears to be reversible owing to the presence of these basal cells (Figure 1C). The lumen of damaged seminiferous tubules was also empty of sperm (Figure 1B), which indicated a decrease in sperm production due to TT tissue damage. On the other hand, extracellular connective tissue containing Leydig cells also showed a decrease in density (Figure 1B, red arrow). All the above‐mentioned pathological tissue changes following the administration of the exosome to normal and physiological tissue changes showed that the lumen thickness increased and became full of sperm. IHC images (Figure 1D–F) revealed high concentration of NF‐kB proteins in TT sections (Figure 1E). This index represented high levels of inflammation in TT animals compared with control (Figure 1D). Also, this index was lower in TT + exosome (Figure 1F) animals than in TT animals.

### 3.6 Apoptotic gene expression

**FIGURE 2:**
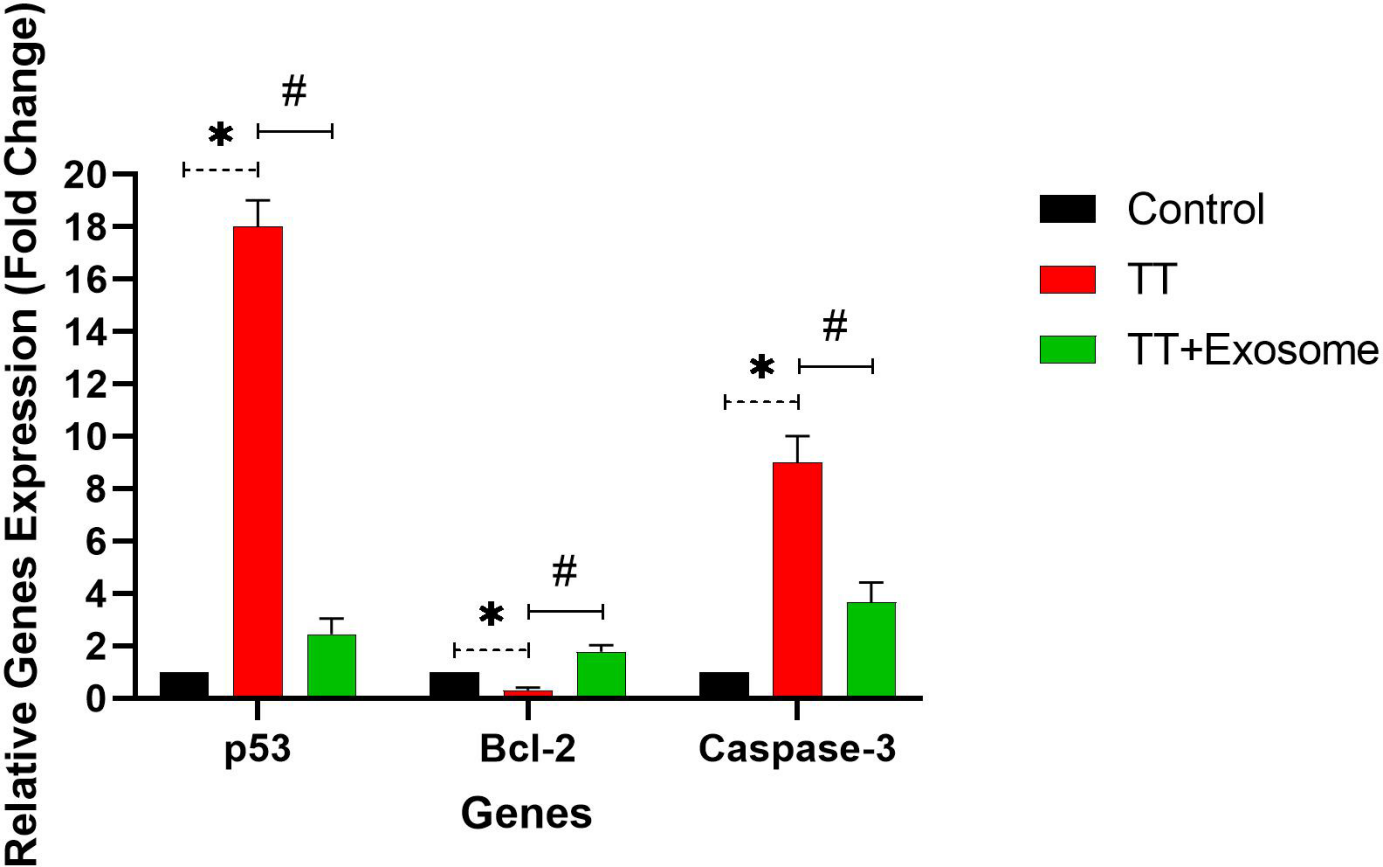
Gene expression of p53, Bcl‐2, and Caspase‐3 control, TT, and TT + exosome groups. *N* = 18 animals in 3 different groups. TT, testicular torsion. * represents significantly (*p* < .05) different from control, and # represents significantly (*p* < .05) different from TT group

Following induction of TT, the apoptotic gene expression of p53 and Caspase‐3 increased significantly (*p* < .05) in TT animals compared with control. Bcl‐2 was decreased significantly (*p* < .05) in TT animals compared with control. Also, these features (p53 and Caspase‐3) were decreased significantly (*p* < .05) following exosome administration in the TT + exosome group compared with TT animals. Bcl‐2 was increased significantly (*p* < .05) following exosome administration in the TT + exosome group compared with the TT group (Figure 2).

## 4 DISCUSSION

In the present animal study, we experimentally demonstrated that exosomes derived from the blood serum of mice have anti‐inflammatory properties that can eliminate tissue inflammation following TT. TT caused testicular damage including seminiferous tubule degeneration and interstitial tissue vanishing due to the high levels of NO and MDA in the tissue. In this situation, the sperm indices were decreased and low‐quality cells were produced. This experiment showed that blood‐derived exosomes had anti‐inflammatory features that restored the pathological state to the physiological state. Following administration of exosomes in TT animals, the levels of NO, MDA, and apoptotic gene expression were decreased and the levels of Tes were restored, leading to production of healthy sperms.

TT is a common pathological condition among men caused by acute twisting of the testicle around the spermatic cord. Thus, the testicular blood vessels are compressed, and blood flow to the testicular tissue is reduced. This condition diminishes the oxygen concentration of testicular tissue; thus, the cellular respiration shifts to anaerobic status.^17^ In this era, a high percentage of reactive oxygen specious is produced and accumulates within the tissue, leading to testicular dysfunction, including low spermatogenesis rate and reduced Tes production. In the absence of urgent treatment, the occurrence of permanent tissue damage is probable.^18^ Another important part of the process of tissue damage due to TT is extensive inflammation of the testicular tissue. Inflammation leads to testicular dysfunction and death due to apoptosis of the spermatogenic lineage. Thus, a wide range of pathological activities occurs, leading to infertility.

The presence of constant blood flow in the tissue leads to a balance in the oxidative‐antioxidant system. Any factor disrupting blood flow to the tissue can increase the oxidative level of the tissue and malfunction of the tissue. Criteria such as nitric oxide and MDA are factors that determine the oxidative level in tissue. As we found in this study, following the onset of TT, blood levels of these 2 factors, which represent tissue oxidative stress, increased dramatically. Abdelzaher and coworkers investigated the effect of dipeptidyl peptidase‐4 (DPP‐4) as well as inhibitors including vildagliptin and sitagliptin, along with nitric oxide synthesis inhibitors. This study was performed on samples with testicular truncation. The results showed that concomitant use of DPP4 inhibitor with nitric oxide synthesis (NOS) inhibitor can reduce the accumulation of oxidative stress and subsequent inflammation. The results of this study clearly show that any factor that reduces the presence of oxidative stress factors can lead to proper cell function.^17^ Testicular tissue damage occurs in emergencies such as TT in 2 important cases: damage to spermatogenic lineage cells and damage to Leydig cells.^2^, ^19^ As we found in this study, damage to spermatogenic lineage cells leads to a decrease in spermatogenesis with abnormal sperm production. Also, by examination of the density of Leydig cells in the extratubular tissue, we observed that the Tes level also was decreased temporarily, considered a sign of damage to these cells available in interstitial tissue.^20^ Yurtcu and his colleagues modeled acute testicular ischemia–reperfusion injury and treatment with melatonin. The results showed that this model of disease, which was similar to that used in our study, could cause tissue damage to the testicular lineage with Leydig cell death. This study showed that, by consuming substances with antioxidant properties, the inflammation can be inhibited and normal tissue activity can be restored.^21^ Finally, it can be concluded that changes in Leydig cell density and decreased activity of spermatogenic lineage cells can lead to extensive tissue changes. As we found in this study, the density of Leydig cells in the extratubular tissue and the arrangement of spermatogenic lineage cells within the tubular space underwent a decrease that resulted in a reduction in sperm production. Also, owing to the decrease in Tes production, the amount of sperm production was low. Tissue staining indicated the presence of NF‐kB proteins as an important landmark in tissue inflammation. Lymphocytic infiltration was also occasionally observed at these times but was not widespread. NF‐kB proteins play an important role in various processes, including inflammation. The presence of these protein classes as transcription factors leads to the expression of different classes of genes involved in inflammation. Therefore, in the presence of this protein, cellular inflammation begins and expands.^22^

## 5 CONCLUSION

Blood‐derived exosomes can act as anti‐inflammatory sources in TT. This study showed that administration of a single dose of exosome immediately after acute testicular injury can significantly reduce TT complications. Our study showed that exosomes can diminish apoptosis and testicular inflammation and lead to the production of high‐quality sperm. It is suggested that, in other experimental studies, the molecular effect of exosomes in the treatment of acute inflammation should be investigated to determine the apparent role of this substance. Thus, the exosome may be suggested as a treatment protocol for the relief of acute inflammation such as TT. For future investigations, it is recommended to use exosomes derived from other body fluids in TT pathologic condition.

## AUTHOR CONTRIBUTIONS

Reza Dadfar conceived and designed the study, supervised the data collection, interpreted the results, and revised the final manuscript. Mona Keivan conducted the data analysis, prepared the tables, and wrote the statistical analysis method and the results. Fatemeh Mansouri Torghabeh and Samira Davoudi conducted the study, collected the data, and organized the dataset. Shima Moradi Maryamneghari assisted in protocol development and drafted the manuscript. All authors approved the final manuscript.

## CONFLICT OF INTEREST

The authors declare that they have no competing interests.
