# Corrigendum

**DOI:** 10.1002/ame2.12288

**Published:** 2022-10-26

In Keivan et al.,^1^ the authors would like to revise the figure 1 as follows,


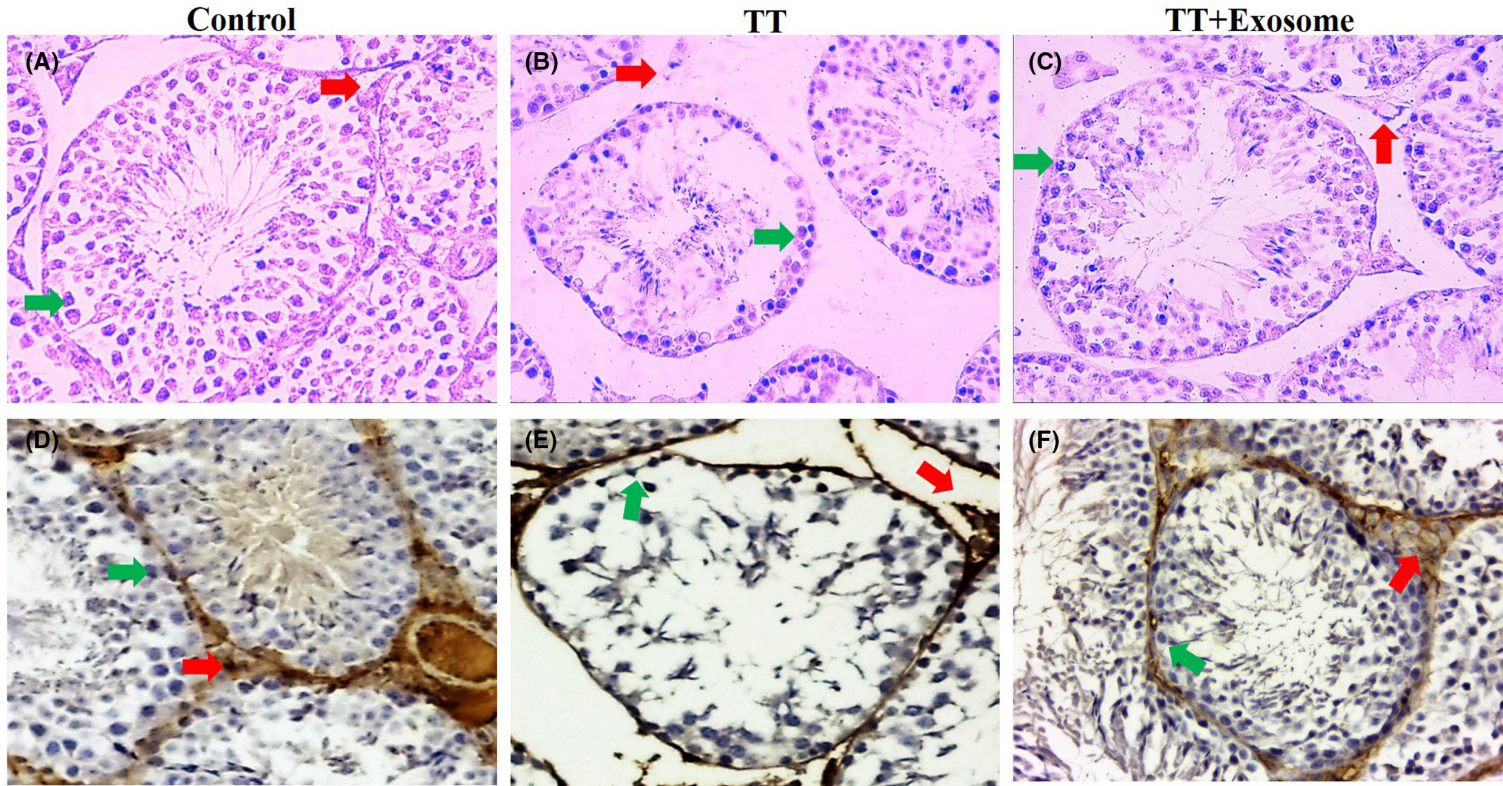


FIGURE 1 Histopathological H&E and IHC images of testis in control (A, D), TT (B, E), and TT + exosome (C, F) groups. Red arrow shows the interstitial connective tissue containing Leydig cell, and green arrow shows basal spermatogenic cell lines. 100× magnification. N = 18 animals in 3 different groups. H&E, hematoxyline and eosin; IHC, immunohistochemistry

Also, the authors would like to revise the graphical abstract and image in online as provided below,


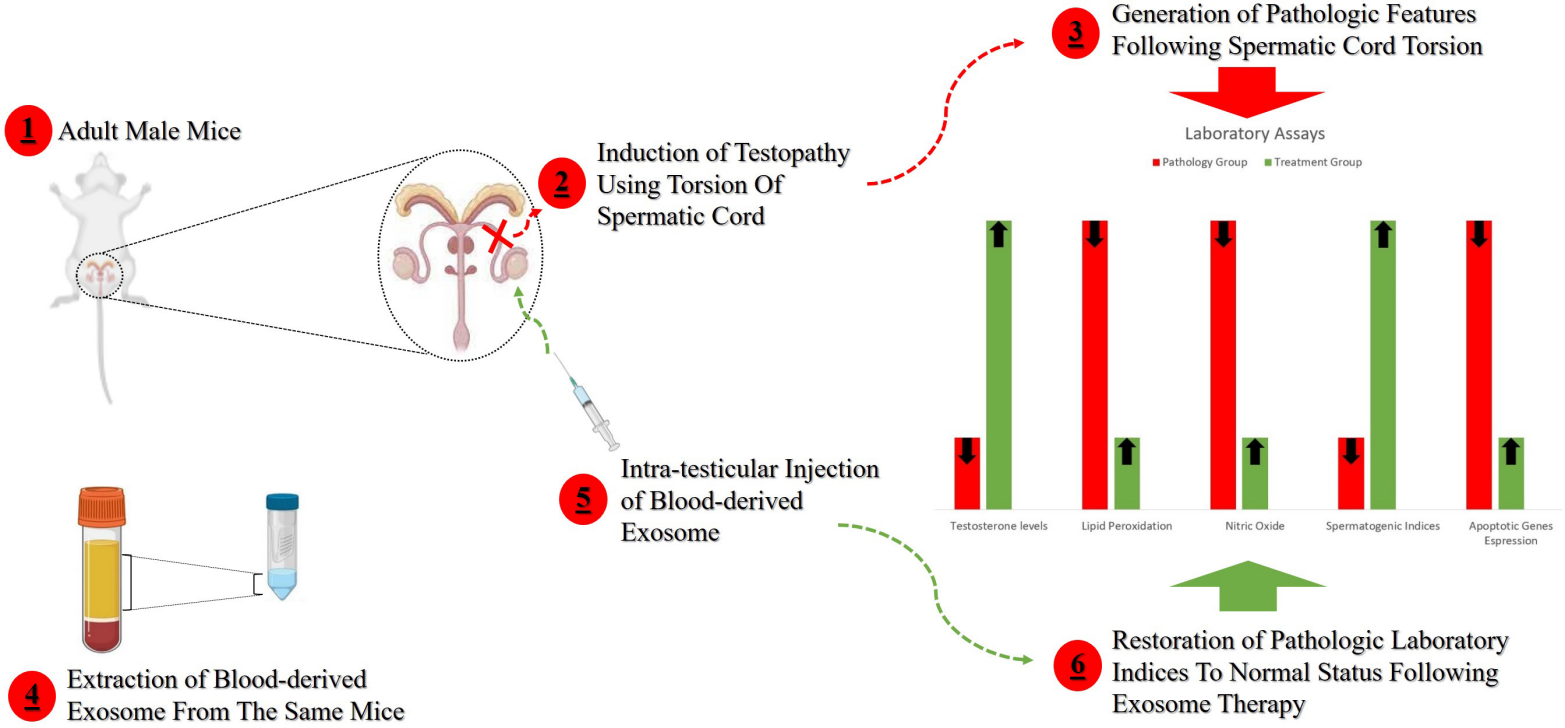


**Graphical abstract**

(1) In this research, adult male mice were used for testopathy modeling. (2) Through temporary torsion of spermatic cord, the laboratory model of testicular torsion was induced. (3) Thus, the pathological condition of testopathy was confirmed by laboratory documentations. (4) Exosomes were derived from mouse blood serum. (5) Blood‐derived derived exosomes were injected into the testis. (6) Finally, the pathological symptoms caused by testicular torsion decreased to a physiological status and sperm production returned to normal conditions.

The Authors apologize for this error.
